# Out-of-pocket health spending among Medicare beneficiaries: Which chronic diseases are most costly?

**DOI:** 10.1371/journal.pone.0222539

**Published:** 2019-09-20

**Authors:** Joelle H. Fong

**Affiliations:** Lee Kuan Yew School of Public Policy, National University of Singapore, Singapore, Singapore; University of Utah, UNITED STATES

## Abstract

**Background:**

Little is known about the impact of different types of chronic diseases on older adults’ out-of-pocket healthcare spending and whether certain diseases trigger higher spending needs than others.

**Methods:**

We use data from the 2014 Health and Retirement Study representing a weighted population of 35,939,270 Medicare beneficiaries aged 65+. Generalized linear models are applied to estimate the effect of different chronic diseases on total out-of-pocket expenditure, adjusted for demographics, socio-economic status, physical health, and other factors. We also decompose total spending by expenditure categories (inpatient, non-inpatient, and prescription drug spending). Sensitivity analysis is performed using a younger sample of older adults aged 50–64.

**Results:**

Cardiovascular disease, diabetes, hypertension and cancer, induce significantly higher adjusted out-of-pocket spending among older adults than other conditions. These results hold regardless how the spending differences are assessed (absolute or percentage terms). For Medicare beneficiaries, cardiovascular disease is associated with an excess out-of-pocket spending of $317 per year, followed by diabetes ($237), hypertension ($150), and cancer ($144). Prescription drug spending is singularly the most important driver of additional expenses for cardiovascular disease, diabetes and hypertension, while non-inpatient services spending accounts for the bulk of increased spending among those with cancer.

**Conclusions:**

Our finding that major noncommunicable diseases impact individuals’ out-of-pocket medical spending differentially–and that service drivers of increased spending may be heterogeneous across disease types–suggest that health professionals and policymakers should recognize that certain chronic diseases exert greater financial toll on the elderly. Interventions to promote more cost efficient healthcare services and consumer choices can help older adults better cope with these expensive long-lasting conditions and reduce the overall burden of noncommunicable diseases.

## Introduction

Noncommunicable diseases (NCDs) are among the most prevalent and costly health conditions in the United States. As of 2013, two out of every three older Americans have two or more chronic health conditions [1,2]. Older adults have higher prevalence of chronic diseases than younger adults. According to nationwide statistics from the American Heart Association (2017), about 85% of Americans aged 65+ have cardiovascular diseases (CVD) as compared to 50% for those aged 45–64. The prevalence of cancers among persons aged 70–79 is nearly double that of those aged 50–59 [3]. Increasing life expectancy over the last decades also implies that there are more people living to old age, possibly with costly chronic health conditions that require ongoing care and management. Medicare is the biggest health insurance program covering the elderly (age 65 and older) in the U.S. and provides financial support for persons who have two or more serious chronic conditions that are expected to last at least a year. Among Medicare fee-for-service beneficiaries, those with NCDs account for 93% of total Medicare spending [4].

Many past studies have highlighted that older adults living with chronic diseases face substantial out-of-pocket (OOP) health expenditures despite Medicare’s near-universal coverage for those aged 65+ [5–10]. Traditional Medicare has relatively high cost-sharing requirements for covered benefits, while certain medical items and services (e.g. routine vision care, hearing exams, and hearing aids) are not covered. Medicare beneficiaries who obtain their benefits through health maintenance organizations (HMOs) face similar financial concerns. Although supplemental coverage through Medigap or employer-sponsored insurance can help lower OOP costs, premiums for these policies can be costly and beneficiaries incur direct expenses in the form of copayments, coinsurance, and deductibles. Financial pressures may discourage some elderly patients from pursuing treatment or result in poor adherence to medications.

In this paper, we assess the impact of chronic diseases on older adults’ OOP medical spending and evaluate whether certain conditions are costlier in terms of additional spending needs. Health service-specific components that drive the increased spending for costly conditions are also identified. We use a nationally representative sample of non-institutionalized, Medicare beneficiaries aged 65+ from the Health and Retirement Study (HRS). A multivariate two-part regression model is implemented to estimate the marginal (incremental) effects of various NCDs, including cancer, diabetes, and CVD, on total spending. For the four costliest conditions in terms of attributable OOP expenditures, we decompose total spending into broad expenditure categories (namely inpatient, non-inpatient, and prescription drug) to identify key drivers of increased spending. This decomposition analysis may be useful for health administrators and policymakers to target interventions.

Although several studies have analyzed the relationship between OOP health spending and chronic diseases, most restrict their analysis to a *single* disease type, e.g. cancer. Langa et al. [5] find that adults age 70+ undergoing cancer treatment spend about $240 more than their counterparts without cancer (who spend approximately $1210 annually); prescription medications account for the bulk of the extra spending. Also focusing on cancer, Davidoff et al. [8] find that Medicare beneficiaries newly diagnosed with cancer face $976 (two-year) excess spending compared to those without cancer. A number of studies focusing on other disease types employ non-elderly samples. For example, Li et al. [11] find that Americans aged 18–64 with diabetes face OOP expenditures exceeding $1,700. Kang and Barner [12] report that increased spending associated with chronic obstructive pulmonary disease is $236±45 per person among adults age 18 and older.

This present study contributes to two strands of health literature. A large body of work has explored the determinants of OOP health expenditures, highlighting the association between OOP spending and age [13]; obesity [14]; health insurance coverage [10,15]; and healthcare reforms such as Medicare Part D implementation [8,16–18]. Many studies also emphasize the important role of chronic illnesses. Nonetheless, these typically had a single-disease focus (as mentioned previously) or used a simple *count* of number of conditions [7,19–20]. For example, Paez et al. [7] find that the estimated mean annual OOP spending is $580 for persons with no NCDs, $870 for those with one NCD, and $1,250 for those with two or more NCDs among persons aged 65–79. In contrast, our study distinguishes across disease *types*. This enables a direct comparison of adjusted spending across chronic diseases so as to pinpoint which ones trigger the largest OOP spending needs. Such insights are valuable for healthcare decision makers and policymakers who are concerned about the financial burden that long-lasting chronic diseases impose on older adults as the population ages.

We also contribute to the broader literature on the disease burden of NCDs. In an era of considerable interest in global health, chronic disease management has raised to the forefront of health policy agendas. Four main disease groups have been identified by the World Health Organization (WHO) and other international health organizations as the leading causes of death and disability in the United States and elsewhere: CVD, cancer, diabetes, and chronic obstructive pulmonary disease. Collectively, these ‘big four’ NCDs pose a significant public health concern due to the disproportionate impact they impose on the disease burden [21–22]. WHO’s ‘big four’ ranking references purely mortality and morbidity indicators. It does not take into account financial dimensions, especially the costs borne directly by individuals out-of-pocket. Accordingly, it is valuable to examine whether the so-called ‘big four’ NCDs are also those which exert the most financial toll on individuals.

The remainder of this paper is organized as follows. The next section describes the HRS data, sample, and variables used in the empirical model. Section 3 outlines the methodology. Section 4 presents the estimation results highlighting the effects of chronic conditions, as well as other factors, on OOP spending. Section 5 extends those results by performing a decomposition analysis to pinpoint sources of excess spending for the four costliest chronic conditions. We also conduct a robustness check using a younger-old sample. Section 6 concludes.

## Methods

### Data and sample

Data are obtained from the 2014 HRS. The HRS is a nationally representative longitudinal survey of U.S. adults over the age of 50, which is conducted by the University of Michigan with funding from the National Institute on Aging. Approximately 20,000 participants are interviewed biennially since 1992, with response rates of more than 85% across waves [23]. Data are de-identified and made publicly available at no charge to users. Our final sample comprises 8,018 non-institutionalized HRS participants 65 years and older who responded to the 2014 interview, and who were enrolled in either fee-for-service Medicare or obtained Medicare benefits through an HMO. We exclude nursing home residents given their distinct patterns of resource use compared with community-dwelling persons [24]. The HRS is based on a stratified multistage area probability sample of U.S. households. The complex sample design, which includes oversamples of Hispanics, Blacks, and households in the state of Florida, requires the use of sampling weights. Individual-level weighting variables are applied to derive nationally representative spending estimates; the weighted population comprises 35,939,270 persons.

### Out-of-pocket health expenditure

Our study uses the individual as the unit of analysis. HRS collates spending information for the following cost categories: hospitalization, nursing home, physician or clinic visits, dental care, outpatient surgery, prescription drugs, home health care, community care, and others. For prescription drugs, respondents are asked, “On average, about how much have you paid out-of-pocket per month for these prescriptions [in the month prior to the interview]?” For all other cost categories, the survey question is phrased, “About how much did you pay out-of-pocket for XX [in the last 2 years]?” To derive respondents’ total annual OOP payment for healthcare, we normalize expenditures for each category to an annual scale then sum across categories. We also group the cost categories by expenditure type: inpatient (hospital stay, hospitalization care, and overnight nursing home use); non-inpatient (physician or clinic visits, outpatient surgery, dental care, in-home medical care and visits by healthcare professionals, community care, and others); and prescription drug.

The HRS uses an innovative “bracketing” method in which respondents who are unwilling to provide an exact amount in response any of the expenditure questions are prompted with response brackets such as, “Did it amount to more than $2000?”; “more than $5000?”; “less than $10 000”; and so on. The information collated from these bracket questions are then used to derive imputed exact expenditure values using a method developed by the RAND Corporation, as described elsewhere [25]. In our sample, imputed responses for bracketed questions are derived for between 0.4% (home care expenditures) and 10% (doctor visits) of respondents for the individual service categories. Our total OOP spending measure reflects self-reported payments for coinsurance, copayments, deductibles, and medically related items and services not covered by insurance. As in Davidoff et al. [8], health insurance premiums are not included because these do not directly relate to medical care use.

### Chronic diseases and other covariates

The HRS collects information on a set of doctor-diagnosed chronic health problems, including heart disease, stroke, cancer, diabetes, chronic lung disease, hypertension, arthritis, and major psychiatric problems. Respondents are asked, “Has a doctor ever told you that you have had a [chronic condition]?”. We construct a dichotomous variable for each chronic disease coded as (0,1), where 1 indicates ever having the condition and 0 otherwise. We combine responses for heart disease and stroke to create a CVD dummy. A total of seven distinct chronic conditions, including the so-called ‘big four’ NCDs, are thus used in our model.

We also include a parsimonious set of control variables widely used by researchers to study the risks associated with OOP health spending [5,8,10]. These include socio-demographic characteristics (sex, race, age, education, marital status); physical and mental health status (functional status limitations, cognitive score, self-reported health); social support factors (whether have children, whether live with others); insurance coverage (Medicaid, private health insurance, private long-term care insurance); and socioeconomic status factors (income and net worth). Medicaid, an insurance program for the indigent, is jointly funded by the federal and state governments, and covers payments for intermediate care facilities, home health care, nursing home care and other residential care services. The inclusion of Medicaid and supplemental insurance variables account for those who have extra insurance to help pay for medical services and items not covered by Medicare, thus lowering OOP spending needs.

To accommodate potential nonlinear age effects, we use categorical age bands (65–74 (ref.), 75–84, 85+). Separate binary markers are constructed to indicate female, non-white, married, high school graduate, had two or more living children, and lived with others in household. Separate indicator variables are also constructed for whether covered by Medicaid, private health insurance, and private long-term care (LTC) insurance. Functional status measures include count of instrumental activities of daily living (IADL) limitations (0 (ref.), 1–3, and 4–5), and count of activities of daily living (ADL) disabilities (0 (ref.), 1–2, and 3+). Self-rated health is a categorical variable (excellent (ref.), very good, good, and fair/poor), while cognitive score is a continuous variable (range from 0–7). Net worth refers to wealth net of liabilities. Wealth is considered in addition to income because elderly persons may draw on accumulated assets to pay for healthcare. In addition, many may have also exited the labour market at ages past 65. We use net worth terciles (low, middle, and high) and annual income quartiles (bottom, second, third, and top).

### Statistical analysis

We use a two-part regression framework to estimate the effects of various chronic disease types on OOP health expenditure. Because a substantial proportion of respondents report not having any OOP spending, a regression using the ordinary least squares method will yield biased results. The two-part regression model accounts for the skewed distribution of the outcome variable and large mass at zero by separating behavior into two stages: the decision to spend, and then the level of spending [26–27]. The first part of the model estimates the probability of observing a positive-versus-zero outcome, whereas the second part estimates the amount of OOP spending conditional on having any. Specifically, we first estimate a probit model for the probability of observing a zero versus positive medical expenditure, and then apply a generalized linear model with log link and gamma distribution in the second part. Separate regressions are performed for total spending, and each service-specific expenditure category (inpatient, non-inpatient, and prescription drug).

About 18% of sampled respondents report not having any form of OOP spending. The median total spending is $600, while mean spending is $1,220. At the 90^th^ percentile, annual expenditure is $3,000. This suggests that the total spending distribution is skewed rightwards with a mass point at zero. The GLM framework [28] implemented in the second part takes into account the right-skewness and avoids potential bias issues in retransformation. Standard specification tests conducted support the use of the log link and the gamma distribution [29–30]. A series of goodness-of-fit tests further confirm that the fitted models do not have significant specification errors. For each outcome variable, we combine the results of the first and second parts to estimate average spending across different disease types. To ensure generalizability of the study findings to the U.S. population, we take into account the complex sample design of the HRS via individual-level sampling weights in all regressions. Statistical analyses are performed using STATA version 14.0 (STATA Corp., TX, USA).

## Results

### Descriptive statistics

Table 1 presents characteristics of the weighted sample. 55% are female, 14% are non-white, 57% are married, and 83% have high school education. The majority (81%) have two or more living children and 71% live with others in the household. Not surprisingly, chronic conditions are quite prevalent among this elderly group: 37% have CVD, 21% have cancer, 26% have diabetes, 69% have hypertension, and 70% have arthritis. Smaller proportions have chronic lung disease (12%) and major psychiatric problem (19%). About two-fifths of respondents (36%) do not have any of the ‘big four’ NCDs (CVD, cancer, diabetes, or chronic lung disease). Among older adults who do have such NCDs, the majority (60%) have only one out of four conditions. About three-quarters of the respondents report good to excellent health and most do not have functional limitations (only 16% have IADL limitations and 19% have ADL limitations). 45% have some form of private health insurance but only 15% have private long-term care insurance. 9% are covered by Medicaid, in addition to Medicare.

**Table 1:** Characteristics of Medicare beneficiaries sample aged 65 and above.

Variables	Mean (SD)
*Main explanatory variables*:	
Cardiovascular diseases, CVD	37%
Cancers	21%
Diabetes	26%
Chronic lung diseases	12%
Hypertension	69%
Major psychiatric	19%
Arthritis	70%
*Other controls*:	
Female	55%
Non-white	14%
High school graduate	83%
Married	57%
Two or more living children	81%
Lives with others	71%
Have Medicaid	9%
Have private health insurance	45%
Have private LTC insurance	15%
Cognition score (scale 0–7)	4.40 (0.89)
*Age*	
65–74 (ref. cat.)	60%
75–84	29%
85+	11%
*ADL disability*	
0 (ref. cat.)	82%
1–2	13%
3+	6%
*IADL limitations*	
0 (ref. cat.)	84%
1–3	13%
4–5	3%
*Self-reported health*:	
Excellent (ref. cat.)	8%
Very good	31%
Good	34%
Fair / poor	27%
*Net worth*	
Low (ref. cat.)	30%
Middle	32%
High	37%
*Yearly Income*	
Bottom quartile (ref. cat.)	22%
Second quartile	23%
Third quartile	26%
Top quartile	29%
*N (unweighted) =*	*8*,*018*
*N (weighted) =*	*35*,*939*,*270*

Source: Author’s calculations based on data from the 2014 Health and Retirement Study. Notes: ADL = activities of daily living; IADL = instrumental activities of daily living; SD = standard deviation (shown only for continuous variables). The sample is weighted using individual-level sampling weights to adjust for the complex sampling design of the HRS survey. All the explanatory variables presented above are indicator (or dummy) variables except for cognition score, age, ADL disability, IADL limitations, self-reported health, net worth and income. For indicator variables, the reference categories are as follows: no CVD, no cancer, no diabetes, no chronic lung disease, no hypertension, no major psychiatric problem, no arthritis, male, white, non-high school graduate, not married, no or one child, live alone, no Medicaid, no private health insurance, and no private LTC insurance. The thresholds for the net worth terciles are <$90,100 (low), $90,100–$367,000 (middle), and >$367,000 (high). The thresholds for the annual income quartiles are <$14,780 (bottom), $14,780–$24,200 (second), $24,200–$41,400 (third), and >$41,400 (high).

Table 2 shows annual total OOP health spending by disease type and expenditure category. Across all diseases, individuals with the condition incur higher mean spending than persons without that condition. For instance persons with and without CVD spend $1,689 and $1,224, respectively, on average, while persons with and without cancer spend $1,616 and $1,337, respectively. The difference in means, e.g. $279 [$1616–1337] for cancer, reflects the increased or excess spending among persons with that disease (shown in bold in Table 2). Before adjusting for any confounding factors, increased spending is largest for CVD ($465), followed by diabetes ($357), chronic lung disease ($323), and finally, cancer ($279). The increased spending of other chronic conditions, e.g. arthritis is $193–270. Excess spending evaluated in percentage terms produce the same ranking. Thus, on an unadjusted basis, the four costliest chronic conditions are identical to WHO’s ‘big four’ NCDs. Unadjusted increased spending for the remaining chronic conditions including hypertension, major psychiatric problem and arthritis are presented in S1 Table.

**Table 2:** Level of annual OOP spending and unadjusted increased spending, by selected chronic condition and expenditure category.

	CVD		Cancer		Diabetes		Lung Disease	
	No	Yes	No	Yes	No	Yes	No	Yes
*n =*	*4*,*940*	*3*,*078*	*6*,*292*	*1*,*726*	*5*,*735*	*2*,*283*	*7*,*046*	*972*
**Total spending**	** **	** **	** **	** **	** **	** **		
Mean^†^	$1,224	$1,689	$1,337	$1,616	$1,305	$1,661	$1,359	$1,682
SD	$1,733	$2,432	$1,937	$2,340	$1,991	$2,125	$2,034	$1,996
Median	$694	$990	$760	$880	$720	$1,000	$755	$1,000
**Increased spending ($)**	** **	**$465**	** **	**$279**	** **	**$356**	** **	**$323**
**Increased spending (%)**	** **	**38%**	** **	**21%**	** **	**27%**	** **	**24%**
*Number of positives*	*4*,*219*	*2*,*674*	*5*,*391*	*1*,*502*	*4*,*992*	*1*,*901*	*6*,*076*	*817*
*As % of n*	*85%*	*87%*	*86%*	*87%*	*87%*	*83%*	*86%*	*84%*
*Breakdown by expenditure category*:								
**Inpatient spending**								
Mean^†^	$1,150	$1,225	$1,067	$1,540	$1,074	$1,484	$1,196	$1,164
SD	$1,862	$2,668	$1,667	$3,582	$1,793	$3,305	$2,478	$1,540
Median	$500	$500	$450	$500	$400	$600	$500	$500
*Number of positives*	*326*	*384*	*526*	*184*	*500*	*210*	*573*	*137*
*As % of n*	*7%*	*12%*	*8%*	*11%*	*9%*	*9%*	*8%*	*14%*
**Non-inpatient spending**								
Mean^†^	$714	$791	$721	$817	$754	$701	$739	$757
SD	$1,272	$1,530	$1,407	$1,213	$1,472	$975	$1,402	$1,052
Median	$300	$350	$300	$375	$300	$340	$300	$375
*Number of positives*	*3*,*600*	*2*,*123*	*4*,*466*	*1*,*257*	*4*,*245*	*1*,*478*	*5*,*083*	*640*
*As % of n*	*73%*	*69%*	*71%*	*73%*	*74%*	*65%*	*72%*	*66%*
**Prescription drug spending**								
Mean^†^	$679	$1,034	$800	$887	$714	$1,092	$785	$1,067
SD	$982	$1,412	$1,134	$1,343	$1,092	$1,357	$1,155	$1,343
Median	$360	$600	$480	$480	$360	$600	$432	$600
*Number of positives*	*3*,*259*	*2*,*274*	*4*,*303*	*1*,*230*	*3*,*909*	*1*,*624*	*4*,*845*	*688*
*As % of n*	*66%*	*74%*	*68%*	*71%*	*68%*	*71%*	*69%*	*71%*

Source: Author’s calculations based on data from the 2014 Health and Retirement Study. Notes: OOP = out-of-pocket; CVD = cardiovascular diseases; SD = standard deviation.

^†^ *P* < .01 for difference in means for OOP spending for persons with versus without a given chronic condition. This difference reflects the increased spending among persons with a given chronic condition. For total OOP expenditures, we present the increased spending (in both dollar and percentage terms) in bold.

Each pair of columns pertain to one of the four major chronic conditions; see text. Alternate pairs of columns are shown in grey background for ease of viewing. Mean and median dollar spending are in nominal U.S. dollars. In addition, for each subgroup analyzed, we report the total number of respondents (‘*n*’), the number of respondents with a positive expenditure (‘*number of positives*’), and the proportion of respondents with a positive expenditure as a percentage of the total (‘*as % of n*’). These rows are shown in italics to differentiate them from dollar amounts. The sample is weighted using individual-level sampling weights to adjust for the complex sampling design of the HRS survey.

Median spending is less than mean spending across all expenditure categories regardless of disease status. This indicates that the OOP expenditure distributions for total, inpatient, non-inpatient, and prescription medication spending are all right-skewed, with relatively few respondents having very large spending. Comparing across the three expenditure categories also provides useful insights. 87% of the 3,078 older adults with CVD report having positive (i.e. at least one dollar of) total OOP spending. However, the proportions of individuals with positive spending in the detailed expenditure categories are smaller (see italicized rows in Table 2). Among the 3,078 older adults with CVD, only 12% incur inpatient spending, 69% incur non-inpatient spending, and 74% incur prescription drug spending. This pattern holds for the other disease types. Of note, in particular, is the observation that more people spend on non-inpatient services and prescription drugs than inpatient services. Yet, older adults who incur inpatient expenses generally spend more. This holds regardless of disease type or status. Average inpatient spending ranges between $1,067 and $1,540, which is considerably higher than the mean expenditures of $679–1,092 reported in the other two expenditure categories.

### Two-part regression results

Table 3 presents the estimation results from the multivariate two-part model using annual total OOP spending. Marginal effects and 95% confidence intervals are reported. We find that almost all the chronic diseases have significant positive impact on spending after adjusting for other important control variables. The size of the effects varies across disease types. Increased spending attributable to CVD is the largest at $317 (95% CI 217–418; *P* < .01). In other words, a Medicare beneficiary with CVD incurs on average $317 more in OOP expenses per year as compared someone without CVD. Additional OOP spending that arise due to cancer ($144, 95% CI 36–252), diabetes ($237, 95% CI 135–339), hypertension ($150, 95% CI 50–250), and arthritis ($131, 95% CI 38–224) are also highly significant (*P* < .01). Chronic lung disease is only mildly significant (*P* < .10).

**Table 3:** Two-part regression model: Increased OOP spending attributable to various chronic conditions accounting for relevant covariates (Medicare beneficiaries aged 65 and above).

Variables	Incremental spending (in $)		95% CI	P value
*Main explanatory variables*:				
Cardiovascular diseases, CVD	317.0	***	(216.6, 417.5)	<0.001
Cancer	144.3	***	(36.1, 252.4)	0.009
Diabetes	237.4	***	(135.4, 339.4)	<0.001
Chronic lung disease	138.7	*	(2.0, 279.4)	0.053
Hypertension	149.7	***	(49.9, 249.5)	0.003
Major psychiatric	85.5		(-36.0, 206.9)	0.168
Arthritis	130.9	***	(38.0, 223.7)	0.006
*Other covariates*:				
Female	116.6	**	(25.0, 208.2)	0.013
Non-white	-119.0	*	(-248.0, 10.0)	0.071
High school graduate	-33.8		(-175.2, 107.6)	0.640
Married	101.3		(-29.4, 232.0)	0.129
Two or more living children	-48.0		(-173.5, 77.5)	0.453
Lives with others	-37.3		(-176.1, 101.5)	0.599
Have Medicaid	-528.1	***	(-661.9, -394.2)	<0.001
Have private health insurance	25.7		(-63.2, 114.6)	0.572
Have private LTC insurance	184.7	**	(42.3, 327.2)	0.011
Cognition score (scale 0–7)	-4.5		(-61.5, 52.6)	0.878
*Age (ref group*: *65–74)*				
75–84	-12.5		(-106.5, 81.5)	0.794
85+	-1.0		(-162.4, 160.4)	0.990
*ADL disability (ref group*: *0)*				
1–2	173.1	**	(23.1, 323.2)	0.024
3+	450.1	**	(96.4, 803.9)	0.013
*IADL limitations (ref group*: *0)*				
1–3	127.1		(-54.0, 308.2)	0.169
4–5	-63.4		(-352.6, 225.7)	0.667
*Self-reported health (ref group*: *excellent)*				
Very good	-93.3		(-302.7, 116.0)	0.382
Good	84.4		(-125.4, 294.2)	0.431
Fair / poor	227.1	**	(0.6, 453.7)	0.049
*Net worth (ref group*: *low)*				
Middle	233.1	***	(121.7, 344.6)	<0.001
High	376.0	***	(251.9, 500.2)	<0.001
*Yearly Income (ref group*: *bottom quartile) *				
Second quartile	181.7	***	(51.2, 312.1)	0.006
Third quartile	195.1	***	(59.5, 330.7)	0.005
Top quartile	374.8	***	(223.1, 526.5)	<0.001

Source: Authors’ calculations based on data from the 2014 Health and Retirement Study. Notes: OOP = out-of-pocket; ADL = activities of daily living; IADL = instrumental activities of daily living. OOP spending refers to total spending per year in nominal U.S. dollars. The sample (*n* = 8,018) is weighted using individual-level sampling weights in 2014 to adjust for the complex sampling design of the HRS survey. Robust standard errors are used to derive the 95% confidence intervals. All the explanatory variables presented above are indicator (or dummy) variables except for cognition score, age, ADL disability, IADL limitations; please refer to Table 1 notes for the reference categories of the indicator variables.

***Level of significance P<0.01

**Level of significance P<0.05

*Level of significance P<0.10

To put these dollar estimates in context, we also evaluate the spending difference in percentage terms. That is, we divide increased spending by the predicted average spending for persons without that disease (results not shown). Our results suggest that Medicare beneficiaries with CVD spend 30.5% more than those without CVD. Those with diabetes spend 20.8% more than their non-diabetes counterparts. Additional spending is 14.1% and 12.4% for hypertension and cancer, respectively. These estimates are higher than those for chronic lung disease (11.8%) and arthritis (11.7%). Thus, on an adjusted basis, the four ‘costliest’ chronic conditions are {CVD, diabetes, hypertension, and cancer}, whether in absolute or relative terms.

Turning attention to the other covariates, we find that female, ADL limitations, poor self-reported health, net worth, and income, are significantly associated with higher aggregate spending. Women spend more than men by about $117 per year (*P* < .05), controlling on all other covariates. Average spending increases with a count of ADL limitations: Medicare beneficiaries with 1–2 limitations spend $173 more, while those with 3+ limitations spend $450 more, as compared to those without ADLs. Persons with fair/poor health incur $227 (*P* < .05) more than those with excellent self-rated health. Both income and assets have significant positive impact on spending, with effects of $233-$376 (*P* < .01) across net worth categories and $182–375 (*P* < .01) across income categories. Finally, we find that Medicaid coverage reduces total spending substantially ($528; *P* < .01). This is not surprising since most state-managed Medicaid programs offer benefits that are not normally covered by Medicare, including nursing home care and personal care services, and hence help lower individuals’ OOP expenses. It is, however, important to note that this significant, negative correlation between Medicaid and OOP spending is evaluated at national-level. It does not account for differences in Medicaid programs across states, including varying criteria on what services are covered, who is eligible, and how physicians and care providers are reimbursed through the program.

### Extensions

To explore which service-specific payments (inpatient, non-inpatient, or prescription drug) drive the excess spending among Medicare beneficiaries with CVD, diabetes, hypertension, or cancer, we estimate the marginal effects by expenditure categories (see Table 4). We find that prescription medication is a major component of the increased OOP spending for Medicare beneficiaries with CVD, diabetes and hypertension in 2014. Pharmaceuticals account for about 90% of the higher spending among beneficiaries with diabetes and hypertension. 67% of the excess OOP expenditure attributable to CVD stems from prescription drugs spending. Additionally, costs of hospitalization care and/or overnight nursing home use (inpatient spending) also contributes significantly to the high costs of care for community-dwelling older adults with CVD. For the fourth costliest chronic condition–cancer–the key driver of increased spending is non-inpatient services (comprising outpatient surgery, doctor visits, dental care, home health care, community care), which accounts for 48% of the total excess spending.

**Table 4:** Sources of increased OOP spending for the four costliest chronic conditions (Medicare beneficiaries aged 65 and above).

Disease	Type of service	Incremental spending (in $)		As % of total
**CVD**	Inpatient	50.3	***	16%
	Non-inpatient	52.9	*	17%
	Prescription drug	206.9	***	67%
	*Total*	*310*.*1*		
**Diabetes**	Inpatient	14.2		5%
	Non-inpatient	5.2		2%
	Prescription drug	242.7	***	93%
	*Total*	*262*.*1*		
**Hypertension**	Inpatient	5.1		3%
	Non-inpatient	14.7		8%
	Prescription drug	159.4	***	89%
	*Total*	*179*.*2*		
**Cancer**	Inpatient	41.3	*	25%
	Non-inpatient	80.8	***	48%
	Prescription drug	45.9		27%
	*Total*	*168*.*0*		

Source: Authors’ calculations based on data from the 2014 Health and Retirement Study. Notes: OOP = out-of-pocket. The sample is weighted using individual-level sampling weights in 2014 to adjust for the complex sampling design of the HRS survey.

***Level of significance P<0.01

**Level of significance P<0.05

*Level of significance P<0.10.

Some studies have raised concerns that chronic disease prevalence and OOP spending have also been increasing among Americans in midlife and early old age [7,31]. To test whether our main results hold for younger-old respondents, we estimate the two-part model for a non-institutionalized sample of adults aged 50–64 in the 2014 HRS (*n* = 6,227). In addition to the controls used previously, we add Medicare to account for individuals under age 65 who qualify for Medicare coverage owing to disabilities or specific medical conditions. Results are shown in Table 5. The findings are largely consistent with those in the main analysis. Marginal effects are significant at the 1% level across various disease types and attributable increased spending is highest for cancer ($510), followed by CVD ($411), diabetes ($350), and hypertension ($307). Thus the four costliest chronic conditions are identical to those derived earlier for the main sample although the exact ranking of diseases differs.

**Table 5:** Two-part regression model: Increased OOP spending attributable to various chronic conditions accounting for relevant covariates (sample aged <65).

Variables	Incremental spending (in $)		95% CI	P value
*Main explanatory variables*:				
Cardiovascular diseases, CVD	410.7	***	(213, 608.4)	<0.001
Cancer	510.1	***	(213.5, 806.6)	0.001
Diabetes	350.1	***	(172.6, 527.6)	<0.001
Chronic lung disease	252.9	*	(-19.7, 525.6)	0.069
Hypertension	307.0	***	(188.3, 425.6)	<0.001
Major psychiatric	292.4	***	(126.9, 457.9)	0.001
Arthritis	177.3	***	(55.1, 299.5)	0.004
*Other covariates*:				
Female	278.4	***	(160.6, 396.3)	<0.001
Non-white	-267.6	***	(-395.3, -139.9)	<0.001
High school graduate	195.9	**	(10.1, 381.7)	0.039
Married	208.9	**	(49.1, 368.8)	0.010
Two or more living children	-151.5	*	(-311.2, 8.1)	0.063
Lives with others in household	-244.3	**	(-454.5, -34.1)	0.023
Have Medicare	86.5		(-162.8, 335.8)	0.496
Have Medicaid	-676.9	***	(-854.8, -499)	<0.001
Have private health insurance	176.3	*	(-0.6, 353.2)	0.051
Have private LTC insurance	-110.4		(-286.7, 66)	0.220
Cognition score (scale 0–7)	-137.4		(-454.5, 179.7)	0.396
*Age (ref group*: *50–54)*				
55–59	175.6		(-47.2, 398.3)	0.122
60–64	208.9	*	(-19.1, 436.8)	0.072
*ADL disability (ref group*: *0)*				
1–2	215.2		(-49.1, 479.5)	0.111
3+	224.8		(-262.3, 711.9)	0.366
*IADL limitations (ref group*: *0)*				
1–3	208.2		(-68.7, 485.2)	0.141
4–5	7.3		(-717.8, 732.4)	0.984
*Self-reported health (ref group*: *excellent)*				
Very good	156.3	**	(0.4, 312.2)	0.049
Good	226.7	***	(64.5, 388.8)	0.006
Fair / poor	683.0	***	(429.7, 936.3)	<0.001
*Net worth (ref group*: *low)*				
Middle	179.8	**	(36.6, 323)	0.014
High	471.7	***	(288.2, 655.3)	<0.001
*Yearly Income (ref group*: *bottom quartile) *				
Second quartile	290.8	***	(98.1, 483.4)	0.003
Third quartile	197.8	**	(4.5, 391.2)	0.045
Top quartile	499.5	***	(263.9, 735.1)	<0.0010

Source: Authors’ calculations based on data from the 2014 Health and Retirement Study. Notes: OOP = out-of-pocket; ADL = activities of daily living; IADL = instrumental activities of daily living. OOP spending refers to total spending per year in nominal U.S. dollars. The sample (*n* = 6,227) is weighted using individual-level sampling weights in 2014 to adjust for the complex sampling design of the HRS survey. Robust standard errors are used to derive the 95% confidence intervals. All the explanatory variables presented above are indicator (or dummy) variables except for cognition score, age, ADL disability, IADL limitations; please refer to Table 1 notes for the reference categories of the indicator variables.

***Level of significance P<0.01

**Level of significance P<0.05

*Level of significance P<0.10

## Discussion

In this study we modeled the effect of various types of chronic diseases on OOP healthcare expenditure among non-institutionalized older adults. Our multivariate two-part analyses revealed that CVD, diabetes, hypertension and cancer, trigger significantly higher spending needs than other diseases such as arthritis. Our results are robust to variations in sample and how spending differences are assessed. Notably, three of the four conditions identified are also listed as WHO’s ‘big four’ NCDs implying that these diseases are not only expensive to society in terms of cost of life, but also exert considerable financial toll on individuals in terms of direct expenses. The costly nature of CVD is perhaps least surprising because stroke and heart failure are currently among the most expensive chronic conditions in the Medicare fee-for-service program [32]. Hypertension, although not a leading cause of death and disability, imposes relatively high OOP costs on older adults, even among those with Medicare coverage. These findings are particularly valuable to policymakers and health professionals seeking to understand the financial dimensions of the growing chronic illness burden in both developed and developing countries [33–34].

Our results underscore the important role of chronic diseases in predicting level of OOP spending among older adults after controlling for other important confounders. Almost all the disease types evaluated have significant positive effects on spending and the magnitudes are relatively large compared to other determinants of spending (e.g. gender). Disaggregating the disease types provided further insights into the heterogeneous impact of various diseases. Among Medicare beneficiaries, the estimated excess spending for the four costliest conditions range from $144–317 per year. In relative terms, beneficiaries with such conditions spend about 12.4–30.5% more than their counterparts without such conditions. Among younger adults age 50–64, the increased spending estimates associated with the four costliest conditions are $307–510. These larger effects can be partially explained by the fact that most persons <65 years are not Medicare-eligible.

Our empirical estimates are comparable to several prior studies. For instance, average spending estimates of $1,300 and $1,160 for persons with and without cancer are consistent with Langa et al. [5] who derived annual expenditures of $1,450 and $1,210 using an earlier wave of the HRS. Our estimated incremental effect of $144 for cancer is within range of past estimates presented ($240–488), but comparatively lower because earlier studies evaluated spending prior to Medicare Part D implementation. Our estimated $243 for additional prescription drug spending among diabetes adults concords with the estimate of $198–233 reported in an earlier study [35]. While the annual increased spending estimates reported here and elsewhere may seem small, it is noteworthy that such excess spending is likely recur year after year because chronic illnesses persist for extended periods. Indeed, many studies have highlighted that the long-term, compounding effects of OOP costs arising from chronic illnesses–together with lost income–can result in severe financial hardship [36–37].

Our analyses were also informative on the health service-specific components that drive increased spending. For three of the four costliest conditions (CVD, diabetes and hypertension), prescription drug spending is singularly the most important driver of additional expenses. These findings are consistent with other recently published studies [38–39], and likely due to the extensive use of prescription drugs in disease management, e.g. oral agents or insulin therapy consumed by diabetes patients. Spending for non-inpatient services accounts for the bulk of increased spending among Medicare beneficiaries with cancer, reflective of the growing trend of outpatient care among cancer patients in the last several years [40–42]. A key conclusion is that service drivers of increased spending may be heterogeneous across disease types. Decomposition analysis can thus help health administrators and policymakers target interventions. For instance, lowering pharmaceutical costs for diabetes through volume purchasing or provider incentives. Value-based insurance design plans, which align individuals’ OOP costs with the value of the health services they receive, can also promote more cost efficient healthcare services and consumer choices [43].

Our study has some limitations which future research can remedy. First, the HRS measures of health status and OOP expenditure data are self-reported. As such, the cost data may be subject to recall bias or incomplete reporting, although in our context, the latter is minimized by the use of the HRS bracketing approach as was described earlier. Second, our evaluation of different disease types is limited to the set of available conditions in the HRS survey. This excludes chronic illnesses such as dementia or viral diseases. Third, the OOP spending for pharmaceuticals we examined is limited to prescription drugs distributed at licensed pharmacies. Information on expenses relating to over-the-counter drugs is not available in the survey. A final limitation is that our analyses are cross-sectional and do not take into account dynamic changes in OOP expenditure over time.

Chronic diseases are on the rise and older adults face the challenge of coping financially with these expensive long-lasting conditions. Recognizing that some chronic conditions may be relatively more costly for individuals in terms of additional OOP spending needs could contribute to more effective targeting of health interventions. Interventions to strengthen early detection and timely treatment of the more costly diseases, for example, can reduce the need for more expensive treatment and excessive OOP spending downstream. Federal and state policies that reduce OOP health expenditure can, in turn, contribute to better health outcomes in the population through improved access and compliance with treatments and thus reduce the overall burden of NCDs to the health ecosystem.

## Supporting information

S1 Table Level of annual OOP spending and unadjusted increased spending, by chronic condition and expenditure category (non-big four conditions). (DOCX)
