# Supplementary material for: Out-of-pocket health spending among Medicare beneficiaries: Which chronic diseases are most costly?
Source: PLoS One. 2019 Sep 20;14(9):e0222539. doi: 10.1371/journal.pone.0222539 (PMC6754145; doi:10.1371/journal.pone.0222539)
Supplement: S1 Table — (DOCX) [file pone.0222539.s001.docx]

**Supplemental Table**

**S1 Table. Level of annual OOP spending and unadjusted increased spending, by chronic condition and expenditure category (non-big four conditions)**

|  | **Hypertension** | | **Major psychiatric problem** | | **Arthritis** | |
| --- | --- | --- | --- | --- | --- | --- |
|  | **No** | **Yes** | **No** | **Yes** | **No** | **Yes** |
| *n=* | *2,289* | *5,729* | *6,591* | *1,427* | *2,291* | *5,727* |
| **Total spending** |  |  |  |  |  |  |
| Mean^†^ | $1,263 | $1,456 | $1,345 | $1,624 | $1,231 | $1,466 |
| SD | $1,827 | $2,116 | $2,035 | $2,005 | $1,800 | $2,119 |
| Median | $650 | $850 | $750 | $970 | $675 | $850 |
| **Increased spending ($)** |  | **$193** |  | **$279** |  | **$235** |
| **Increased spending (%)** |  | **15%** |  | **21%** |  | **19%** |
| *Number of positives* | *1,952* | *817* | *5,693* | *1,200* | *1,914* | *4,979* |
| *As % of n* | *85%* | *14%* | *86%* | *84%* | *84%* | *87%* |
|  |  |  |  |  |  |  |
| *Breakdown by expenditure category:* | | |  |  |  | |
| **Inpatient spending** |  |  |  |  |  |  |
| Mean^†^ | $1,357 | $1,137 | $1,170 | $1,260 | $1,123 | $1,212 |
| SD | $2,093 | $2,396 | $2,440 | $1,889 | $1,308 | $2,574 |
| Median | $550 | $400 | $500 | $500 | $700 | $400 |
| *Number of positives* | *154* | *556* | *545* | *165* | *161* | *549* |
| *As % of n* | *7%* | *10%* | *8%* | *12%* | *7%* | *10%* |
| **Non-inpatient spending** |  |  |  |  |  |  |
| Mean^†^ | $785 | $720 | $731 | $790 | $741 | $741 |
| SD | $1,243 | $1,427 | $1,404 | $1,193 | $1,475 | $1,320 |
| Median | $330 | $300 | $300 | $363 | $300 | $338 |
| *Number of positives* | *1,738* | *3,985* | *4,765* | *958* | *1,618* | *4,105* |
| *As % of n* | *76%* | *70%* | *72%* | *67%* | *71%* | *72%* |
| **Prescription drug spending** | |  |  |  |  |  |
| Mean^†^ | $671 | $872 | $779 | $987 | $660 | $880 |
| SD | $1,006 | $1,236 | $1,165 | $1,241 | $865 | $1,280 |
| Median | $360 | $480 | $420 | $600 | $360 | $480 |
| *Number of positives* | *1,334* | *4,199* | *4,535* | *998* | *1,457* | *4,076* |
| *As % of n* | *58%* | *73%* | *69%* | *70%* | *64%* | *71%* |

Source: Author’s calculations based on data from the 2014 Health and Retirement Study.

Notes: OOP = out-of-pocket; CVD = cardiovascular diseases; SD = standard deviation.

^†^ $P$ <.01 for difference in means for OOP spending for persons with versus without a given chronic condition. This difference reflects the increased spending among persons with a given chronic condition. For total OOP expenditures, we present the increased spending (in both dollar and percentage terms) in bold.

Each pair of columns pertain to one of the three chronic conditions; see text. Alternate pairs of columns are shown in grey background for ease of viewing. Mean and median dollar spending are in nominal U.S. dollars. In addition, for each subgroup analyzed, we report the total number of respondents (‘*n*’), the number of respondents with a positive expenditure (‘*number of positives*’), and the proportion of respondents with a positive expenditure as a percentage of the total (‘*as % of n*’). These rows are shown in italics to differentiate them from dollar amounts. The sample is weighted using individual-level sampling weights to adjust for the complex sampling design of the HRS survey.
